# Prevalence of potential drug − drug interactions in the cardiothoracic intensive care unit patients in a Chinese tertiary care teaching hospital

**DOI:** 10.1186/s40360-022-00582-6

**Published:** 2022-06-14

**Authors:** Haitao Wang, Haitao Shi, Na Wang, Yan Wang, Li Zhang, Yujie Zhao, Jiao Xie

**Affiliations:** 1 Department of Pharmacy, The Second Affiliated Hospital of Xi’an Jiaotong University, Xi’an, China; 2 Department of Gastroenterology, The Second Affiliated Hospital of Xi’an Jiaotong University, Xi’an, China; 3 Department of Intensive Care, The Second Affiliated Hospital of Xi’an Jiaotong University, Xi’an, China

**Keywords:** Drug − drug interaction, Risk factor, Intensive care unit, Drug interaction database, Adverse drug event

## Abstract

**Background:**

With an increasing number of reviews describing clinically significant drug–drug interactions (DDIs), the scope and severity of interactions involving commonly used drugs in cardiothoracic intensive care units (CCUs) remain unclear. This study aims to identify risk factors and determine the incidence of potential DDIs in intensive care units.

**Methods:**

DDIs were identified based on the profile of the prescribed drug and classified according to the Micromedex drug interaction database. Potential risk factors associated with DDIs have been identified.

**Results:**

A total of 3193 medication episodes were evaluated, and 680 DDIs (21.3%) were found. A total of 203 patients were recruited into the study, with an average of 3.4 DDIs per patient [95% confidence interval (3.2 − 3.6)]. A total of 84.2% of the patients experienced at least one DDI. Anticoagulant and antiplatelet agents were involved in 33.5% (228/680) of the potential drug − drug interactions in the CCU. Univariate analysis and multiple logistic regression analysis showed that the age of the patient and the number of medications prescribed were significantly correlated with the occurrence of DDIs. In multiple linear regression analysis, the number of DDIs had a significant correlation only with the number of prescription drugs.

**Conclusions:**

A high prevalence of DDIs was observed, especially in intensive care units without pharmacist intervention and computerized drug monitoring systems, highlighting the need for active surveillance to prevent potential adverse events.

## Background

A drug–drug interaction (DDI) is defined as a medication response to the administration of two or more drugs that differs from the expected pharmacological effect [1]. In clinical practice, potential DDIs can cause adverse effects with varying effects from treatment failure to serious adverse drug events (ADEs).

Patients in the intensive care unit (ICU) routinely receive a large number of drugs, which are associated with DDIs, highlighting the need to study these interactions in these settings [2–4]. Studies have shown that 10 − 16% of all preventable adverse events among ICU patients were DDI-related, and approximately 5% of all ICU patients were likely to experience an ADE during hospitalization due to a DDI [5, 6]. By establishing a risk profile, reducing risk and preventing side effects can improve patient safety [7–9].

Currently, DDI identification software is available, and manual identification by clinical pharmacists helps detect and prevent the occurrence of DDIs [10, 11]. However, physicians in some developing countries still need to identify DDIs based on clinical experience. DDIs are one of the leading causes of adverse drug events that can compromise patient safety [12]. Therefore, identifying the incidence, increasing awareness of risk factors for the potential occurrence of DDIs, and familiarity with the mechanisms involved in DDIs can markedly reduce the incidence of drug interactions in hospitalized patients.

Studies evaluating the prevalence of potential drug interactions have been conducted in patients in the intensive care unit; however, data describing the potential DDIs that occur in the CCU are still lacking in China. Differences in drug treatment options in different regions may lead to changes in the prevalence of DDIs, especially in the absence of clinical pharmacist intervention and the assistance of identification software. This study aims to analyze the risk factors, frequency and types of DDIs in CCUs in a Chinese university − affiliated teaching hospital, which will significantly guide future clinical drug selection and patient safety.

## Methods

A cross-sectional study was performed in the CCU (15 beds) of our tertiary care teaching hospital. All prescriptions and clinical information for patients admitted to the CCU were collected in the hospital information system; however, medication surveillance was not conducted by the system. Clinical pharmacists did not participate in the treatment during the study period, and DDI alerts were not available for CCU patients. Adult patients who were admitted to the CCU for more than 24 h and prescribed at least two medications between July 2018 and December 2020 were included. The study protocol was approved by the Ethics Committee of the Second Affiliated Hospital of Xi’an Jiaotong University. All medical data and patient demographic data were collected from the electronic hospital information system, including prescribing information, sex, age and length of stay in the CCU.

A DDI was defined as a possible interaction between two or more drugs that could result in a change in the therapeutic effect and/or the toxicity of one or both drugs [13]. All patient prescriptions for medications were scanned for DDIs utilizing the Micromedex drug interaction database. The database identifies interacting drugs, mechanisms, severity, reliability (documentation) rating (E = excellent, G = good, F = fair), potential outcomes, and clinical management. Depending on the severity of the interaction, the drugs are divided into four categories: minor, moderate, major and contraindicated. The frequency of DDIs was calculated as the number of DDIs per patient; the number of patients who experienced at least one DDI were included. The types of DDIs were described by listing the 10 most frequently occurring DDIs [14].

In this study, the interactions classified as moderate, important, or contraindicated were considered clinically relevant. Univariate and multivariate logistic regression analyses were performed to identify risk factors associated with the occurrence of clinically relevant DDIs (minor interactions were removed from the analysis of risk factors considering the limited clinical relevance). The dependent variable was the incidence of at least one clinically relevant DDI per patient. Models for accurately predicting the occurrence of DDIs were calibrated using three versions of *R*^*2*^: the Hosmer and Lemshow *R*^*2*^, based on the chi − square score; Cox and Snells’ *R*^*2*^, based on the deviance of the model; and Nagelkerke’s *R*^*2*^, which provides a correction. Variables with bivariate *p* values less than 0.1 were analyzed using a multivariate logistic regression model. The statistically significant associations and plausible variables were included in a multivariate regression model using the enter method to control confounding effects [15]. Additionally, multiple linear regression analysis was performed using the number of clinically relevant interactions per patient as the dependent variable. Statistical analysis was performed using SPSS statistical package version 19.0 (IBM SPSS Inc., Chicago, IL, USA).

## Results

### Patient demographic and clinical information

A total of 203 patients were included in the study; the average age was 63.7 years, and the average length of stay in the CCU was 6.18 days. During admission to the CCU, 171 patients (84.2%) experienced at least one DDI, while the frequency was 3.4 DDIs per patient (95% CI 3.2 − 3.6) in this subgroup of patients. Among the patients, 131 (55.1%) were male, and 72 (44.9%) were female. The number of prescribed medications was 15.73 ± 7.18 (Table 1).

**Table 1:** Demographic and characteristics of CCU patients

Characteristics	Values
Number of patients (n)	203
Age (mean ± SD)	63.66 ± 13.87
Gender	
*Male*	131
*Female*	72
Number of drugs per prescription (mean ± SD)	15.73 ± 7.18
Length of stay in the CCU in days (mean ± SD)	6.18 ± 2.92
Severity of pDDIs per patients	
*Contraindicated*	4
*Major*	255
*Moderate*	360
*Minor*	61

### Prevalence of DDIs

During the course of the study, 680 DDIs were identified, quantified, classified, and distributed with 106 combinations of prescribed drugs. A large number of potential DDIs were detected, including contraindicated DDIs of 4 pairs, a majority of 255 pairs, a moderate number of 360 pairs and a minor number of 61 pairs (Table 1). The most common potential drug interactions included the 10 pairs of interacting drugs shown in Table 2, representing 22.5% (153/680) of all observed potential drug interactions identified in the database. In all identified DDIs, anticoagulant and antiplatelet agents were involved in 33.5% (228/680) of potential interactions in the CCU.

**Table 2:** The ten most frequently occurring potential drug-drug interactions in the cardiovascular intensive care unit

Drug combination	Severity	Frequency	Potential adverse events
aspirin/clopidogrel	Major	22	Increased risk of bleeding
aspirin/nitroglycerin	Moderate	21	Increased nitroglycerin concentrations and additive platelet function depression
aspirin/insulin	Moderate	19	Increased risk of hypoglycemia
methylprednisolone/insulin	Moderate	17	Increased risk of bleeding or diminished effects of warfarin
aspirin/metoprolol	Moderate	15	Increased blood pressure
digoxin/torsemide	Moderate	13	Increased risk of digoxin toxicity (nausea, vomiting, cardiac arrhythmias)
candesartan/potassium chloride	Moderate	13	Increased risk of hyperkalemia
metoprolol/lidocaine	Major	12	Increased risk of lidocaine toxicity (anxiety, myocardial depression, cardiac arrest)
morphine/ticagrelor	Major	11	Increased morphine exposure
moxifloxacin/amiodarone	Major	10	Increased risk of QTc prolonging effects

### Association with predicting factors

In this study, 619 clinically relevant drug interactions were included in the analysis of factors excluding the 61 minor pairs. In the univariate analysis, the incidence of DDIs was significantly associated with the number of prescribed medications, length of hospital stay, and patient age (Table 3). However, no association was observed between sex and incidence of DDIs (*p* = 0.82). Variables with a univariate *p* value less than 0.1 were analyzed using a multivariate logistic regression model. Patient age [OR = 1.04; 95% CI (1.01 − 1.07)] and the number of prescribed drugs [OR = 1.20; 95% CI (1.07 − 1.33)] were found to be strongly related to DDIs [*R*^*2*^ = 11.9 (Hosmer–Lemeshow), 0.19 (Cox-Snell), 0.31 (Nagelkerke)]. Additionally, factors associated with the number of interactions per patient were investigated using univariate and multiple linear regression (Table 4). For the multivariate linear regression analysis, sex, patient age, number of drugs prescribed, and length of hospital stay were selected based on the univariate regression analysis. The adjusted results showed a significant positive relationship between the number of clinically relevant DDIs and the number of drugs prescribed.

**Table 3:** Univariate and multivariable-adjusted analyses of factors associated with the occurrence of DDIs

Variable	Unadjusted OR (95% CI)	Unadjusted *p* value	Adjusted OR (95% CI)	Adjusted *p* value
Gender (reference female)	1.09 (0.51 to 2.35)	0.82	-	-
Age	1.05 (1.02 to 1.07)	< 0.0001	1.04 (1.01 to 1.07)	**0.004**
No. of prescribed drugs	1.23 (1.13 to 1.35)	< 0.0001	1.20 (1.07 to 1.33)	**0.001**
No. of hospitalization days	1.35 (1.15 to 1.57)	< 0.0001	1.06 (0.87 to 1.31)	0.56

Hosmer- Lemeshow *R*^*2*^ = 11.9; 8 df; *p* = 0.16; Cox and Snells’ *R*^*2*^ = 0.19; Nagelkerke’s *R*.^*2*^ = 0.31

**Table 4:** Univariate and multivariable-adjusted analyses of factors associated with the number of DDIs

Variable	Coefficients	Unadjusted *p* value	Coefficients	Adjusted *p* value
Gender (reference female)	-0.58 (-1.26 to 0.09)	0.09	-0.55 (-1.10 to 0.002)	0.05
Age	0.03 (0.01 to 0.05)	0.003	0.02 (-0.002 to 0.03)	0.08
No. of prescribed drugs	0.18 (0.15 to 0.22)	< 0.0001	0.21 (0.16 to 0.25)	** < 0.0001**
No. of hospitalization days	0.17 (0.1 to 0.24)	< 0.0001	-0.71(-0.15 to 0.009)	0.08

*R*^*2*^ = 0.37; Corr. *R*^*2*^ = 0.36; F (df = 4; 420.7) = 29.3

## Discussion

In our study, a total of 3193 medication episodes were evaluated, 680 DDIs were found in the CCU, and anticoagulant and antiplatelet agents were involved in 33.5% of the potential drug interactions. Univariate analysis and multiple logistic regression analysis showed that age and the number of medications prescribed were significantly correlated with the occurrence of DDIs. The number of DDIs had a positive correlation with the number of prescribed drugs.

Previous studies have reported a relatively low prevalence rate of 40% − 79.5%, and in our study, 84.2% of CCU patients developed at least one relevant DDI [7, 16–19]. Contextual variables, including availability of CPOE and/or CDSS and the presence of clinical pharmacists on daily rounds, may explain the high variability in the reported prevalence of DDIs. In addition, international differences in drug availability may contribute to regional variations in the number of DDIs [20].

Patients taking multiple drugs in our study had a higher risk of DDIs (*p* < 0.001), consistent with previous findings in which multiple drugs predisposed patients to adverse effects of drug therapy such as DDIs [18, 21]. Patients may in turn be exposed to a higher probability of DDIs when prescribed an increased number of drugs. A positive relationship was found between age and the incidence of DDIs; that is, older patients were more likely to develop DDIs. It is generally accepted that the older a patient is, the greater the risk of developing DDIs when the number of drugs is increased. This is because increasing age correlates with chronic disease, which is diagnosed over time. Two studies involving cardiovascular disease patients in Pakistan and Iran also found that older populations were at higher risk of DDIs [18, 22]. The risk of adverse events due to DDls was higher in the elderly due to age-related physiological changes affecting the pharmacokinetics and pharmacodynamics of various drugs [23]. Surprisingly, geriatric patients were not identified as a determinant for the number of clinically relevant DDIs. This may be attributed to the fact that older age and the number of concomitant medications are usually highly correlated in multivariate analyses.

Sex may be a risk factor for DDIs in some studies, although in our study, we found that sex was not significantly associated with the incidence and the number of DDIs. A study of ICU patients found a significant association of DDIs with male, whereas another study identified female as a factor associated with the development of DDIs [24, 25]. There is no credible evidence for sex differences, which may be due to physiological and pharmacological reasons.

Drug − induced excessive QT prolongation can increase the risk of torsades de pointes. There are three general mechanistic classifications that include producing an additive QTc prolonging effect, enhancing antiplatelet or anticoagulant effects and involving the cytochrome P450 enzyme system [17]. Studies have shown that patients with prolonged QT intervals may have an increased length of hospital stay, which is associated with higher all − cause mortality [26]. Although there are many mechanisms leading to QT prolongation, including those with genetically related long QT syndrome, bradycardia, or hypokalemia, drug therapy, particularly DDI, is one of the major causes of these adverse events [27, 28]. The results from a single center study showed that up to 70% of adverse events related to QT prolongation in critically ill patients were due to drug interactions [29]. The drugs most commonly used with these DDIs in our study include moxifloxacin, amiodarone, and fluconazole. These medications are known to be associated with QT prolongation, and torsades de pointes is perhaps the most risky occurrence identified with QT prolongation. It is important to be aware of the adverse effects and risks of taking these drugs, especially when given numerous prescriptions known to affect the QT interval.

Not surprisingly, anticoagulant and antiplatelet agents were the most widely recognized interacting drug groups in our results. Anticoagulant and antiplatelet drugs (e.g., warfarin, clopidogrel and acetylsalicylic acid) are important therapeutic agents in the CCU for the treatment of cardiovascular diseases. However, when these medications are administered in combination with nonsteroidal anti − inflammatory drugs, significant DDIs may occur, which may increase the risk of bleeding [30, 31]. These extended risks may outweigh the consequences of the risks associated with each drug. Therefore, the therapeutic risk − benefit profile of these treatments needs to be explored. One of the most frequent drug interactions occurs with the concurrent use of aspirin and clopidogrel. These drugs enhance toxicity through pharmacodynamic synergy, increasing the threat of bleeding. However, a growing number of older people are using low-dose aspirin in combination with clopidogrel or ticlopidine to prevent atherosclerotic events (ischemic heart disease, ischemic stroke and peripheral arterial disease). Therefore, when coadministration is necessary, careful monitoring of blood counts and signs and symptoms of bleeding is necessary.

The limitation of this study was that a comprehensive review of all potential adverse events resulting from DDIs was not performed. Although measuring the clinical outcomes of DDIs makes sense in theory, it is almost impossible to attribute all clinical treatment outcomes of critically ill patients to DDIs. Another limitation is that only one database was used for evaluation, which may lead to incomplete drug information.

## Conclusions

The incidence of DDIs is high in CCU patients in our hospital and is negatively affected by age and the number of prescribed drugs, which highlights the need for active surveillance to prevent potential adverse events caused by DDIs. The participation of clinical pharmacists in multidisciplinary teams and embedded surveillance software with electronic prescription databases can help isolate DDIs and minimize the occurrence of associated risks.

## Data Availability

The data that support the findings of this study are available on request from the corresponding author. The data are not publicly available due to patient privacy or ethical restrictions.
